# Six years genotype distribution of Human Papillomavirus in Calabria Region, Southern Italy: a retrospective study

**DOI:** 10.1186/s13027-017-0154-5

**Published:** 2017-07-27

**Authors:** Luisa Galati, Cinzia Peronace, Maria Teresa Fiorillo, Rosanna Masciari, Cristina Giraldi, Salvatore Nisticò, Pasquale Minchella, Vincenzo Maiolo, Giorgio Settimo Barreca, Nadia Marascio, Angelo Giuseppe Lamberti, Aida Giancotti, Maria Gabriella Lepore, Francesca Greco, Maria Vittoria Mauro, Annelisa Borelli, Giuseppa Lo Bocchiaro, Giovanni Surace, Maria Carla Liberto, Alfredo Focà

**Affiliations:** 1 Institute of Clinical Microbiology, Department of Health Sciences, “Magna Graecia” University, Viale Europa, 88100 Catanzaro, Italy; 2 Unit of Microbiology and Virology, Polo Sanitario Nord ASP 5, Reggio Calabria, Italy; 3 Unit of Mirobiology and Virology, “Pugliese-Ciaccio” Hospital, Catanzaro, Italy; 4 Unit of Microbiology and Virology, “Annunziata” Hospital, Cosenza, Italy; 5 Unit of Microbiology and Virology, General Hospital, Lamezia Terme, Italy; 6 Unit of Microbiology and Virology, “G. Jazzolino” Hospital, Vibo Valentia, Italy

**Keywords:** HPV, Epidemiology, Genotypes distribution, Calabria Region

## Abstract

**Background:**

Although analysis of the Human papillomavirus (HPV) genotype spread in a particular area has a crucial impact on public health and prevention programmes, there is a lack of epidemiological data regarding HPV in the Calabria region of Italy. We therefore update information on HPV age/genotype distribution by retrospectively analysing a cohort of women, with and without cervical lesions, living in Calabria, who underwent HPV DNA testing; moreover, we also evaluated HPV age/genotype distribution in a subset of patients with cervical lesions.

**Methods:**

Cervical scrape specimens obtained from 9590 women (age range 20–75 years) from January 2010 to December 2015 were tested for HPV DNA. Viral types were genotyped by Linear Array HPV Genotyping® test (Roche, USA) at the Clinical Microbiology Operative Unit of six hospitals located in four provinces of the Calabria region.

Cervical scrape specimens were also used to perform Pap smears for cytological analysis in a subset of 405 women; cytological classification of the samples was performed according to the Bethesda classification system.

**Results:**

A total of 2974 women (31%) (C.I. 95% 30.09–31.94) were found to be HPV DNA positive for at least one (57.3%) or several (42.7%) HPV genotypes. Of single genotype HPV infections, 46.5% and 36.4 % were classed as high-risk (HR, Group 1) and low-risk (LR, Group 3) respectively, while 16.9% were classed as probably/possibly carcinogenic and 0.2% undetermined risk. Stratified by age, total HPV distribution, showed the highest prevalence within the range 30–39 years (37.2%), while single genotype infection distribution displayed a peak in women from the age range 20–29 years (37.5%). The most common high-risk HPV type was HPV 16 (19.1%), followed by HPV 31 (9.1%).

**Conclusions:**

We provide epidemiological data on HPV age/genotype distribution in women living in the Calabria region with or without cytological abnormalities, further to the enhancement of HPV screening/prevention programmes for the local population.

## Background

Human papillomavirus (HPV) infection of the anogenital tract is the most common sexually transmitted infection; it has a broad range of clinical manifestations, ranging from subclinical and self-limited to persistent and associated with malignant progression. [1].

The mucosal HPV genotypes, grouped into the genus alpha papillomavirus, have been classified into high-risk (HR HPV) and low-risk types (LR HPV), according to their malignant potential [2]. HPV16 and HPV18 are known to be powerful carcinogens, but an International Agency for Research on Cancer (IARC) working group has classified a total of twelve, evolutionarily linked HPV genotypes (16, 18, 31, 33, 35, 39, 45, 51, 52, 56, 58, and 59) as presenting a high risk of carcinogenesis to humans (Group I). Based upon their phylogenetic relatedness, other types were classified as possible (Group 2B) or probable carcinogens (Group 2A) [3].

HR HPV types are a well-established cause of cervical cancer, and there is a growing body of data on the role of HR HPV in other anogenital cancers (anal, vulval, vaginal, penile), as well and head and neck cancers [4, 5].

In Italy, cervical cancer is the third most common female cancer in women aged 15 to 44 years, and, according to the Ministry of Health, each year about 3,200 new cases of cervical cancer are diagnosed with 1,500 women dying from this cancer [6]. Epidemiological studies, carried out in different Italian regions [7–9], on the prevalence of HPV in women with normal cervix or cytological abnormalities have confirmed HPV 16 as the most frequent high-risk genotype identified in all neoplastic and non-neoplastic conditions.

However, several studies have reported heterogeneous HPV-type distributions in different geographical areas [10, 11]. This depends on a complex interaction between viral molecular characteristics and host immunity, as well as the sexual behaviours and age of the target population. It is therefore essential for the distribution of type-specific HPV prevalence to be analysed in each geographical area in order for efficacious public awareness and prevention programmes to be developed.

Indeed, cervical cancer control consists of primary prevention by vaccination, and secondary prevention through screening to diagnose and treat precancerous cervical lesions. However, adherence to screening in the Calabria region is lower than the national standard (respectively 33% and 39%, steps 2010–12) [12], and therefore a region-wide public information campaign is urgently required.

With this in mind, we set out to collect epidemiological data on the prevalence of HPV infection and age/genotype distribution in women who underwent HPV DNA testing in several centres across the region. Moreover, we also evaluated HPV age/genotype distribution in a subset of patients with cervical lesions.

## Methods

### Study population and sample collection

Cervical scrape specimens were obtained from 9590 women (age range 20–75 years) presenting to six hospitals located in four provinces of Calabria region, southern Italy: Catanzaro (University *"Magna Graecia"* Hospital, *"Pugliese-Ciaccio"* Hospital, and *"Giovanni Paolo II"* Hospital), Reggio Calabria (*"Polo Sanitario Nord Azienda Sanitaria Provinciale 5"*) Cosenza (*“Annunziata”* Hospital) and Vibo Valentia *(“G. Jazzolino”* Hospital), during the period 2010–2015. All specimens were tested for HPV DNA at the hospitals’ respective Clinical Microbiology Operative Units.

For all samples DNA extraction was performed by an automated method using EasyMag (bioMérieux); eluted DNA was used for Polymerase Chain Reaction (PCR) amplification of a 450-bp fragment from the L1 HPV region, using primer PGMY09/11, by Linear Array HPV Genotyping Test (LA) (Roche, USA) [13]. An additional primer pair targets the human β-globin gene to provide a control for cell adequacy, extraction and amplification. HPV genotypes were detected by hybridization using a reverse line blot system with type-specific probes for simultaneous detection of 37 HPV genotypes. Negative and positive controls were provided with the kit and used in each test.

Results of molecular procedures were interpreted according to the manufacturer’s instructions and IARC classification. Viral isolates were classified into: 12 HR HPV (HPV 16, 18, 31, 33, 35, 39, 45, 51, 52, 56, 58, 59), 13 LR HPV (HPV6, 11, 40, 42, 54, 55, 61, 62, 72, 81, 83, 84 and CP6108), 1 probably carcinogenic (HPV 68), 7 possibly carcinogenic genotypes (HPV 26, 66, 53, 67, 70, 73, 82) and 4 genotypes for which the risk is still undetermined (HPV 64, 69, 71 and IS39). In 405 women with or without cervical lesions, cervical scrape specimens were also used to perform Pap smear for cytological analysis in order to evaluate HPV age/genotype distribution in this subset. Cytological findings were classified according to the Bethesda classification system as follows: negative for intraepithelial lesions and malignancy (NILM), atypical squamous cells of undetermined significance (ASC-US), low-grade squamous intraepithelial lesions (L-SIL) and high-grade squamous intraepithelial lesions (H-SIL).

The study was designed as a retrospective analysis. The design was approved by the Catanzaro University Hospital Ethics Committee in compliance with the Declaration of Helsinki.

### Statistical analysis

Statistical analysis was carried out on GraphPad Prism version 6.07 to establish the percentage values and their confidence intervals (CI 95%). The odds ratio (OD) value was determined using the online calculator at: https://www.medcalc.org/calc/odds_ratio.php. [14]. The OD value was used to establish the association between ASC-US, L-SIL/NILM and specific HPV groups. In all findings a *p* value <0.05 was considered statistically significant.

## Results

### Overall HPV prevalence

Out of the 9590 women tested, a total of 2974 (31%) (95% CI 30.09–31.94) were found to be HPV DNA positive for at least one (57.3%) or several (42.7%) HPV genotypes. Single infections from HR HPV Group 1, specifically HPV 16, 18, 31, 33, 35, 39, 45, 51, 52, 56, 58 or 59, were observed in 792 women (46.5% of those infected) (95% CI 44.09–48.82); infections with LR HPV Group 3 or indeterminate risk, specifically HPV 6, 11, 40, 42, 54, 55, 61, 62, 72, 81, 83, 84; CP6108, 71; or IS39, were detected in 625 women (36.6%) (95% CI 34.40-38.97), while HR HPV Group 2A/2B infections, specifically HPV 26, 66, 53, 67, 70, 73, 82 or 68, were observed in 288 women (16.9%) (95% CI 15.19–18.74).

Stratified by age, single genotype distribution displayed a peak in women of the age range 20–29 years (37.5%) (95% CI 35.27–39.86), while total HPV infections showed the highest prevalence in the range 30–39 years (37.2%) (95% CI 35.47–38.94). Indeed, multiple infection distribution showed the highest prevalence value in the age class 30–39 years (39.2%) (95% CI 36.52–41.88) (Table 1).

**Table 1:** Overall HPV prevalence. Distribution of single and multiple infections in 2974 women, stratified by age

Age classes (years)	Single HPV infections		Multiple HPV infections		Total HPV infections	
	No.	%	No.	%	No.	%
20-29	640	37.5	320	25.2	960	32.3
30-39	609	35.7	497	39.2	1106	37.2
40-50	320	18.8	369	29.1	689	23.2
>50	136	8	83	6.5	219	7.3
Overall	1705	100%	1269	100%	2974	100%

Considering the prevalence of HPV genotypes grouped according to the three available vaccine formulations, the prevalence of HPV 16 and HPV 18, present in the bivalent vaccine, amounted to 22.7% of the overall infections, while the prevalence of HPV 16, 18, 31, 33, 45, 52, 58, 6 and 11, whose viral antigens are covered by the nonavalent vaccine, was 58.6%. Moreover, in young women between 20 and 39 years of age, the overall prevalence of bivalent (HPV 16, 18), quadrivalent (HPV 16, 18, 6, 11) and nonavalent viral types (HPV 16, 18, 31, 33, 45, 52, 58, 6 and 11) was 8.7%, 15.7% and 23.1%, respectively. In women aged 40+, the prevalence was 4.1% for bivalent HPV vaccine types, 6.7% for quadrivalent types and 9.5% for nonavalent types.

**Fig. 1:**
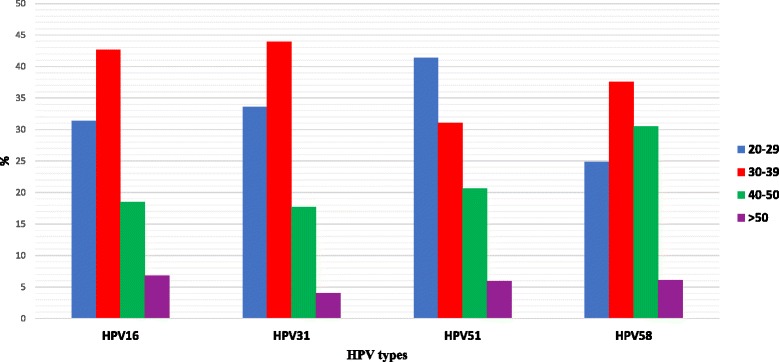
HR HPV genotypes stratified by age classes. Prevalence of the most common HR HPV types (HPV 16, 31, 51, 58) stratified by age classes

Concerning the prevalence of infections with the most representative HPV genotypes, we observed possibly carcinogenic single genotype infections accounting for 16% of overall HPV infections, and, in particular, possibly high-risk (pHR) HPV 53 exhibited a prevalence of 12.3% in multiple infections (Table 2). In single HPV infections, on the other hand, the four most common HR HPV types were HPV 16, 31, 51 and 58, with a prevalence of 16.4%, 6.8%, 4.2% and 3.7%, respectively. Stratification of the most common HR HPV types by age revealed a peak prevalence for HPV 16 and HPV 31 in the 30–39 year age group; HPV 51 was mainly detected in women aged 20–29 while HPV 58 was mostly found in the 30–39 and 40–50 age groups (Fig. 1). As a whole, these findings support the necessity for vaccination of young women in order to prevent HR HPV infections.

**Table 2:** HPV genotype distribution. Prevalence of infections by the most representative HPV genotypes

HPV genotype	Number of infections per genotype (*n*)	HPV prevalence in the overall population (%) (*n*=9590)	Prevalence in HPV positive women (%) (*n*=2974)	Prevalence in single HPV infections (%) (*n*=1705)	Prevalence in multiple HPV infections (%) (*n*=1269)
16	569	5.9%	19.1%	280 (16.4%)	289 (27.7%)
31	270	2.8%	9.1%	116 (6,8%)	154 (12.1%)
51	204	2.1%	6.8%	72 (4.2%)	132(10.4%)
18	108	1.1	3.6%	38 (2.2%)	70 (5.5%)
33	78	0.8%	2.6%	17 (1.0%)	61 (4.8%)
45	83	0.9%	2.8%	36 (2.1%)	47 (3.7%)
52	158	1.6%	5.3%	36 (2.1%)	122 (9.6%)
58	197	2.1%	6.6%	63 (3.7%)	134 (10.6%)
11	35	0.4%	1.2%	15 (0.9%)	20 (1.6%)
6	246	2.6%	8.3	94 (6.1%)	152 (12%)
53	263	2.7%	8.8%	107 (6.2%)	156 (12.3%)
73	159	1.6%	5.3%	59 (3.4%)	100 (7.8%)
66	196	2%	6.6%	76 (4.5%)	120 (9.4%)

### HPV infections and cervical lesions

In a subset of 405 women with or without cytologically demonstrated cervical lesions, analysed between January to December 2015, we also evaluated HPV status/age distribution according to cervical lesion. Accordingly, women (median age 39.7 years; age range 17–75) were classified as NILM (151/405), ASC-US (147/405), L-SIL (97/405) or H-SIL (10/405). The overall percentage of HPV DNA status was 34.8% (141/405) (95% CI 30.34–39.58), and HPV infection was found to be significantly associated with cervical lesions (OR 5.4% 95% CI 3.2–9.2, p<0,0001). Indeed, single infection with any type of HPV was detected in 53.9% (76/141) (95% CI 45.68-61.92) of cases (Table 3).

**Table 3:** HPV distribution across different cervical cytological statuses. Prevalence of HPV positivity in single and multiple infections, according to cytological cervical lesion

Cytological diagnosis	HPV DNA (*n*=141) Single Infection *n* (%)	Multiple Infections *n* (%)
NILM	10 (13.1)	11 (16.9)
ASC-US	36 (47.5)	23 (35.4)
L-SIL	27 (35.5)	28 (43.1)
H-SIL	3 (3.9)	3 (4.6)
TOTAL (*n*=141)	76 (53.9%)	65 (46.1%)

In this subset of patients, 50% (38/76) (95% CI 39.03-60.97) of those exhibiting a single infection, tested positive for a high-risk Group 1 HPV, 25% (19/76) (95% CI 16.63-35.78) for a low-risk Group 3 or undetermined risk, and 25% (19/76) (95% CI 16.63-35.78) for Group 2A/2B types respectively. The overall HPV prevalence increased with lesion severity: NILM (14.5%), ASC-US (39.4%), L-SIL (56.7 %) and H-SIL (60%).

When the women’s age was matched with cervical lesions and HPV status, we found that the mean age of women with negative concordant status for HPV DNA plus cytological analysis was 39.3, whereas the mean age of HPV DNA-positive women with negative cytology was 32.3 years. The mean age of women with both Pap test and HPV positivity was 38.6, and the mean age of women with positive Pap test plus negative HPV DNA was 42.3. Multiple HPV infections were detected in 46.1% (65/141) (95% CI 38.08–54.32) of overall HPV infections in this subset (Table 3).

Further analysis showed that high-risk Group 1 prevalence increased in women with ASC-US (9.6%) and L-SIL (13.1%), as compared with women displaying normal cytology (4.9%), while HR HPV Group 2A/2B frequencies in women with ASC-US, L-SIL and normal cytology were 4.4%, 4.7% and 1.2% respectively. In addition, HR Group 2A/2B types showed a stronger association with L-SIL (OR 11.7, 95% CI 4.1–33.2) than HR Group 1 (OR 8.1, 95% CI 4.3–15.1) and LR Group 3 types (OR 4.7, 95% CI 2.3–9.8).

**Fig. 2:**
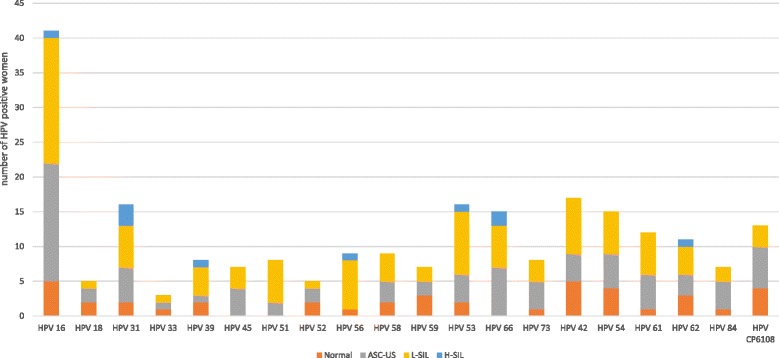
HPV genotypes according to cervical lesions. Number of findings of the most common HPV genotypes detected in 141 positive women, according to cytological status

Concerning HPV genotype distribution according to cervical lesions, HPV 16 was the most frequent high-risk genotype, identified in 41/141 HPV positive women (overall infections 29.1%) (95% CI 21.58–36.32), of which 17/59 women had ASC-US lesions (28.8%) and 18/55 women L-SIL (32.7%). In addition, the prevalence of HPV 18 in overall infections was 3.5% (5/141) and the most common HPV genotypes in H-SIL was HPV 31, which was detected in 50% of cases (3/6) (Fig. 2).

## Discussion

To counter the lack of epidemiological data on HPV in Calabria (to our knowledge this is the first of such studies), we gathered data on the prevalence of HPV infection and age/genotype distribution in women attending six different hospitals in the region. This multicentre analysis revealed a total prevalence of HPV infection of 31.0 %, which, as per the available literature on the subject, varied according to age [15]. In particular, we detected the highest prevalence of single infections (37.5%) in women aged 20–29. This is in line with the results of several studies that have shown a greater prevalence of HPV in younger women, followed by a reduction of HPV infections with increasing age [16, 17]. That being said, we also found a peak of total, as well a multiple, infections in the 30–39-year age group (37.2%), which may reflect local sexual behavioural trends (e.g. number of partners).

Regarding the different HR HPV genotypes in the tested population, we detected several that were most represented in both single and multiple infections, namely HPV 16, 31, 51 and 58. HPV 18, a type strongly associated with cervical cancer, was detected in 3.6% of women. Our results are similar to those recently reported by Veldhuijzen et al., in which two European female populations were tested in HPV screening trials [18]. Comparable results were also obtained in Sardinia, a major Italian island, during a study on the female population exhibiting cervical cytological abnormalities [19]. Also, our data are consistent with those previously reported on HPV types prevalence and distribution in Italian population [8, 9].

The high prevalence of HPV 16 in both single and multiple infections, mainly in 30–39 age group, confirms the widespread distribution of this virus. It is also important to note that HPV 18, the second most common high-risk HPV types in cervical cancer, was rather high in the 30–39 age group, even though its prevalence was very low (3.6%) across the overall population [20–22]. Another important finding was the distribution of HPV types not covered by current vaccines. Among these, HPV 51, 53, 73 and 66 were observed to have a high prevalence among the women analysed in our sample. As a whole, these observations may be of interest to those working on vaccination and local HPV prevention schemes.

In particular, the coverage of the HPV vaccine schedule in the Calabria region is very low (ranging from 46.36% to 70.88 % of girls born in the period from 1997 to 2003) [23]; increasing HPV-vaccination coverage, both across the local population and in terms of HPV targeting, therefore represents a major public health issue. In this regard, our findings underscore the importance of keeping up-to-date information on HPV distribution, both to monitor vaccine efficacy and the spread of the disease.

## Conclusions

Our epidemiological data on the HPV age/genotype distribution in the Calabria region of Italy indicate the need to improve the local HPV screening plan, whose efficacy should be closely monitored in order to maximise efficacy while minimising the cost to the local health service.

## Abbreviations

HPV: Human papillomavirus
HR: High risk
IARC: International agency for research on cancer
LA: Linear array
LR: Low risk
PCR: Polymerase chain reaction

## References

[1] Ignacio G, Bravo IG, Felez-Sanchez M (2015). Papillomaviruses Viral evolution, cancer and evolutionary medicine.

[2] IARC monographs on the evaluation of carcinogenic risks to humans: A review of human carcinogens: part B. Biological agents. 100th edition. Lyon, France: International Agency for Research on Cancer. 2011. p.261-319. http://monographs.iarc. fr/ENG/Monographs/vol100B/mono100B-11.pdf.

[3] Clifford GM, Howell-Jones R, Silvia Franceschi. Judging the carcinogenicity of human papillomavirus types by single/multiple infection ratio in cervical cancer .Int. J. Cancer. 2011; DOI: 10.1002/ijc.25833. 10.1002/ijc.25833 21140454

[4] Tommasino M. The human papillomavirus family and its role in carcinogenesis. Semin Cancer Biol. 2013; 10.1016/j.semcancer.2013.11.002 10.1016/j.semcancer.2013.11.002 24316445

[5] Nagayasu E, Kiyofum E, Griffin H, Doorbar. Human Papillomaviruses; Epithelial Tropisms, and the Development of Neoplasia. Viruses. 2015; doi:10.3390/v7072802. 10.3390/v7072802 PMC4517131 26193301

[6] ICO Information Centre on HPV and Cancer (HPV Information Centre). Human Papillomavirus And Related Diseases Report, ITALY. 2017. www.hpvcentre.net.

[7] Baussano I, Franceschi S, Gillio-Tos A, Carozzi FM, Confortini M, Dalla Palma P, De Lillo M, Del Mistro A, De Marco L, Naldoni C, Pierotti P, Schincaglia P, Segnan N, Zorzi M, Giorgi-Rossi P, Ronco G. Difference in overall and age-specific prevalence of high-risk human papillomavirus infection in Italy: evidence from NTCC trial. BMC Infect Dis. 2013; doi:10.1186/1471-2334-13-238. 10.1186/1471-2334-13-238 PMC3669053 23706168

[8] Rossi PG, Bisanzi S, Paganini I, Di Iasi A, Angeloni C, Scalisi A, Macis R, Pini MT, Chini F, Carozzi FM. HPV Prevalence Italian Working Group. Prevalence of HPV high and low risk types in cervical samples from the Italian general population: a population based study. BMC Infect Dis. 2010; doi:10.1186/1471-2334-10-214. 10.1186/1471-2334-10-214 PMC2916912 20646310

[9] Ronco G, Ghisetti V, Segnan N, Snijders PJ, Gillio-Tos A, Meijer CJ, Merletti F, Franceschi S. Prevalence of human papillomavirus infection in women in Turin, Italy. Eur J Cancer. 2005, 2005; 10.1016/j.ejca.2004.07.005 10.1016/j.ejca.2004.07.005 15661556

[10] De Sanjosé S, Diaz M, Castellsagué X, Clifford G, Bruni L, Muñoz N, Bosch FX. Worldwide prevalence and genotype distribution of cervical human papillomavirus DNA in women with normal cytology: a meta-analysis. The Lancet Infectious Diseases. 2007; DOI: 10.1016/S1473-3099(07)70158-5. 10.1016/S1473-3099(07)70158-5 17597569

[11] Tornesello ML, Duraturo ML, Botti G, Greggi S, Piccoli R, De Palo G, Montella M, Buonaguro L, Franco M (2006). Buonaguro, and The Italian HPV Working Group. Prevalence of Alpha-Papillomavirus Genotypes in Cervical Squamous Intraepithelial Lesions and Invasive Cervical Carcinoma in the Italian Population. Journal of Medical Virology..

[12] De Biase GA, Azzarito C, Bianchi C, De Luca A, Gullà D, Lopresti S, Macchione D, Mignuolo D, Rizzo L, Zappia F. Screening Programs for Cervical Cancer: Investigation “SWOT” in Calabria Journal of Pharmacy and Pharmacology. 2016; doi: 10.17265/2328-2150/2016.06.007.

[13] Steinau M, Onyekwuluje JM, Scarbrough MZ, Unger ER, Dillner J, Zhou T. Performance of Commercial Reverse Line Blot Assays for Human Papillomavirus Genotyping. J Clin Microbiol. 2012; doi:10.1128/JCM.06576-11. 10.1128/JCM.06576-11 PMC3347105 22357500

[14] Lydia S. Murdiyarso, Melissa Kartawinata, Iffat Jenie, Grace Widjajahakim, Heriawaty Hidajat, Ruth Sembiring, I. Made Nasar, Santoso Cornain, Farid Sastranagara, Ahmad Rusdan Handoyo Utomo. Single and multiple high-risk and low-risk Human Papillomavirus association with cervical lesions of 11,224 women in Jakarta*.* Cancer Causes Control. 2016; doi: 10.1007/s10552-016-0816-4. 10.1007/s10552-016-0816-4 27752850

[15] Howell-Jones R, de Silva N, Akpan M, Oakeshott P, Carder C, Coupland L, Sillis M, Mallinson H, Ellis V, Frodsham D, Robinson TI, Gill ON, Beddows S, Soldan K. Prevalence of human papillomavirus (HPV) infections in sexually active adolescents and young women in England, prior to widespread HPV immunisation. Vaccine. 2012; doi:10.1016/j.vaccine.2012.04.006. 10.1016/j.vaccine.2012.04.006 22516212

[16] De Sanjose S, Almirall R, Lloveras B, Font R, Diaz M, Muñoz N, Catala I, Meijer CJ, Snijders PJ, Herrero R, Bosch FX. Cervical human papillomavirus infection in the female population in Barcelona. Spain. Sex Transm Dis. 2003; doi:10.1097/01.OLQ.0000080177.82204.E0. 10.1097/01.OLQ.0000080177.82204.E0 14520179

[17] Agarossi A, Ferrazzi E, Parazzini F, Perno CF, Ghisoni L. Prevalence and type distribution of high-risk human papillomavirus infection in women undergoing voluntary cervical cancer screening in Italy. J Med Virol. 2009; doi:10.1002/jmv.21347. 10.1002/jmv.21347 19152401

[18] Veldhuijzen NJ, Berkhof J, Gillio-Tos A, De Marco L, Carozzi F, Del Mistro A, Snijders PJ, Meijer CJ, Ronco G. The Age Distribution of Type-Specific High-Risk Human Papillomavirus Incidence in Two Population-Based Screening Trials. Cancer Epidemiol Biomarkers Prev. 2015; doi:10.1158/1055-9965.EPI-14-0628. 10.1158/1055-9965.EPI-14-0628 25300476

[19] Meloni A, Pilia R, Campagna M, Usai A, Masia G, Caredda V, Coppola RC. Prevalence and molecular epidemiology of human papillomavirus infection in italian women with cervical cytological abnormalities. J Public Health Res. 2014; doi:10.4081/jphr.2014.157. 10.4081/jphr.2014.157 PMC4140382 25170506

[20] Wheeler CM, Hunt WC, Cuzick J, Langsfeld E, Pearse A, Montoya GD, Robertson M, Shearman CA, Castle PE; New Mexico HPV Pap Registry Steering Committee. A population- based study of Human papillomavirus genotype prevalence in the United States: baseline measures prior to mass human papillomavirus vaccinations. Int J Cancer. 2013; doi: 10.1002/ijc.27608. 10.1002/ijc.27608 PMC3852415 22532127

[21] Monsonegro J, Zerat L, Syrjanen K, Zerat JC, Smith JS, Halfon P. Prevalence of type specific Human papillomavirus infection among women in France: implications for screening, vaccination, and a future generation of multivalent HPV vaccine. Vaccine. 2012; doi:10.1016/j.vaccine.2012.06.013. 10.1016/j.vaccine.2012.06.013 22713720

[22] Monsonego J, Cox JT, Behrens C, Sandri M, Franco EL, Yap PS, Huh W. Prevalence of high-risk human papillomavirus genotypes and associated risk of cervical precancerous lesions in a large U.S. screening population: data from the ATHENA trial. Gynecol Oncol. 2015; doi:10.1016/j.ygyno.2015.01.551. 10.1016/j.ygyno.2015.01.551 25667973

[23] http://www.epicentro.iss.it/problemi/hpv/aggiornamenti.asp. Up-to-date at December 31, 2015.

